# Cascaded Kerr photon-blockade sources and applications in quantum key distribution

**DOI:** 10.1038/s41598-017-07589-8

**Published:** 2017-08-04

**Authors:** Ao Li, Yiheng Zhou, Xiang-Bin Wang

**Affiliations:** 1 State Key Laboratory of Low Dimensional Quantum Physics, Tsinghua University, Beijing, 100084 People’s Republic of China; 2 Synergetic Innovation Center of Quantum Information and Quantum Physics, University of Science and Technology of China, Hefei, Anhui 230026 China; 3 Jinan Institute of Quantum Technology, Shandong Academy of Information and Communication Technology, Jinan, 250101 People’s Republic of China

## Abstract

To raise the repetition rate, a single-photon source based on Kerr quantum blockade in a cascaded quantum system is studied. Using the quantum trajectory method, we calculate and simulate the photon number distributions out of a two-cavity system. A high quality single-photon source can be achieved through optimizing parameters. The designed photon source is further applied to the decoy state quantum key distribution (QKD). With and without statistical fluctuation, the key rate can be both raised drastically.

## Introduction

Single-photon sources as indispensable tools have been widely used in different quantum tasks including quantum optics, quantum communications and so on. Specifically, in the decoy state quantum key distribution (QKD)^1–6^, a secure key can be generated with imperfect single-photon sources^7–9^. To obtain high key rate in QKD, one needs both a high quality single-photon source and a high repetition rate of the source. To realize high quality single-photon sources, the quantum blockade process is a rather promising way^10,11^.

Single-photon blockades have been realized in different systems, such as single emitter (atom and quantum dot) systems^12–15^ and nonlinear (Kerr) medium^16^. And the Kerr photon blockade refers to the happening of single-photon blockade in a cavity with Kerr-type medium. These experiments have already demonstrated photon antibunching and sub-poissonian distribution. Through using a cavity, one can acquire high efficiency in collecting single photons from the blockade. Also, it has been demonstrated that an ordinary Kerr-type material can produce very large effective nonlinear susceptibility, which allows strong interaction among different photons. Besides this, Kerr systems do not require any precise positioning^17,18^. Nevertheless, the repetition rate of Kerr cavity is limited. Specifically, limited by the cavity linewidth, the repetition rate of output light pulse is limited to sub GHz in the Kerr photon blockade system^19^.

In this work, we propose a cascaded method for Kerr photon blockade systems. This proposal is to enhance the potential repetition rate of single-photon sources based on the principle of single-photon blockade in a single mode cavity with Kerr-type nonlinear response. Through cascading cavities, we find an enhanced probability of single photon occupancy, and simultaneously a reduced vacuum and multi-photon probability, which allows to relax the constraints on the repetition rate imposed by the cavity lifetime. Thus our proposed method can improve both the repetition rate and the single-photon quality. Particularly, we use the quantum trajectory method which is based on the evolution of a Monte Carlo wave function (MCWF) of small systems^20–22^ and simulate the output photon number distributions in two-cavity systems. Then we apply such quasi-sources to the decoy-state QKD and we find the key rate can be raised drastically.

## Results

### Model for cascaded cavities

**Figure 1:**
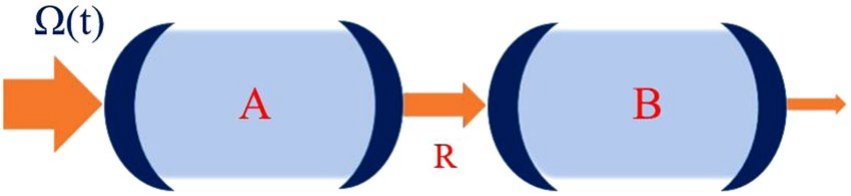
A cascaded Kerr photon blockade system under a coherent driven pulse.

We start with a compound quantum system with two cavities A and B. Cavity B is driven by the radiated emittion from cavity A. The two cavities are cascaded and mediated by a reservoir R (see Fig. 1). Each of the cavities has a single-mode field inside within a Kerr-type medium. The free Hamiltonians of cavity A and B including interactions only inside cavities are^23–25^ (in this letter we set *ħ* = 1):

1 $${H}_{a}={{\rm{\Delta }}}_{a}{a}^{\dagger }a+{\chi }_{a}{({a}^{\dagger })}^{2}{a}^{2},$$

2 $${H}_{b}={{\rm{\Delta }}}_{b}{b}^{\dagger }b+{\chi }_{b}{({b}^{\dagger })}^{2}{b}^{2}.$$

Here, *a*^†^(*b*^†^), *a*(*b*) are the creation and annihilation operators for cavity mode A (B), and Δ = Δ_*a*_ = Δ_*b*_ is the detuning between the center frequency of the driving pulse and the resonator. The nonlinearity strength *χ*_*a*_(*χ*_*b*_) is proportional to the real part of the third-order nonlinear susceptibility, depending on the nonlinear material and the mode volumes of the resonator.

The total Hamiltonian of the system can be divided into parts: the free Hamiltonian of cavity A and B including interactions only inside cavities; the interaction between cavity A or B and the reservoir R; the Hamiltonian of R. That is:

3 $$H={H}_{a}+{H}_{b}+{H}_{R}+{H}_{aR}+{H}_{bR},$$

The interaction Hamiltonians are^26^:

4 $${H}_{aR}={\sqrt{\kappa }}_{a}(ia{\varepsilon }^{\dagger }(0)+{\rm{H}}{\rm{.c}}.),$$

5 $${H}_{bR}={\sqrt{\kappa }}_{b}(ib{\varepsilon }^{\dagger }(l)+{\rm{H}}{\rm{.c}}.).$$

where *κ*_*a*_ (*κ*_*b*_) denotes the cavity decay of cavity A (B). Operators *ε*^†^(0) and *ε*^†^(*l*) stand for the fields that couple to cavity A and B. They are written in photon flux units. The travel distance of photons from cavity A to B is *l*. The distance *l* is small enough and *t*_0_ = *l*/*c* ≈ 0. Under the Born-Markov approximation^27^, photon can be only annihilated from A and created in B, while the reverse process cannot happen. At the same time, operators *ε*(0) and *ε*(*l*) have the relation:

6 $$\varepsilon (l)={U}_{a}^{-1}({t}_{0})(\varepsilon (0)+\frac{1}{2}\sqrt{{\kappa }_{a}}a){U}_{a}({t}_{0}),$$

or

7 $$\varepsilon (0)={U}_{a}({t}_{0})\varepsilon (l){U}_{a}^{-1}({t}_{0})-\frac{1}{2}\sqrt{{\kappa }_{a}}a.$$

where the operator *U*_*a*_ is defined as ref.^28^:

8 $${U}_{a}({t}_{0})=\exp (i({H}_{a}+{H}_{b}+{H}_{aR}){t}_{0}).$$

One can compute the properties of the light filed from cavity A first. Then one computes the cavity-B part. However, the computation is qutie complex. So we consider coupling the two cavities into S. And the total Hamiltonian of the system is

9 $$H={H}_{S}+{H}_{R}+{H}_{SR}.$$

where *H*_*S*_ represents the coupled cavities and *H*_*SR*_ represents the interaction between the cavity and the reservoir:

As photons travels from A to B in *t*_0_ = *l*/*c*, the retarded density operator of the system is also defined as:

10 $${\rho }_{{\rm{ret}}}\equiv {U}_{a}({t}_{0})\rho {U}_{a}^{-1}({t}_{0}).$$

So *H*_*bR*_ should be revised as:

11 $${({H}_{bR})}_{{\rm{ret}}}=\sqrt{{\kappa }_{b}}(ib{{\varepsilon }^{\dagger }}_{{\rm{ret}}}(l)+{\rm{H}}{\rm{.c}}.).$$

where

12 $${\varepsilon }_{{\rm{ret}}}(l)={U}_{a}({t}_{0})\varepsilon (l){U}_{a}^{-1}({t}_{0}).$$

Thus we can finally obtain

13 $${({H}_{bR})}_{{\rm{ret}}}=i\sqrt{{\kappa }_{b}}(b\varepsilon {(0)}^{\ast }+\frac{1}{2}\sqrt{{\kappa }_{a}}{a}^{\dagger }b-\frac{1}{2}\sqrt{{\kappa }_{a}}a{b}^{\dagger }-{b}^{\dagger }\varepsilon (0)).$$

and

14 $${H}_{S}={H}_{a}+{H}_{b}+i\sqrt{{\kappa }_{a}{\kappa }_{b}}(\frac{1}{2}{a}^{\dagger }b-\frac{1}{2}a{b}^{\dagger }).$$

The interaction term *H*_*SR*_ is:

15 $${H}_{SR}=(\sqrt{{\kappa }_{a}}a+\sqrt{{\kappa }_{b}}b){\varepsilon }^{\dagger }(0)+{\rm{H}}{\rm{.c}}.$$

So the reduced density operator *ρ*_*ab*_ of the coupled system S satisfies the Master equation:

16 $$\frac{d{\rho }_{ab}}{dt}=-i[{H}_{S},{\rho }_{ab}]+\frac{1}{2}(2{C}_{2}{\rho }_{ab}{{C}_{2}}^{\dagger }-{{C}_{2}}^{\dagger }{C}_{2}{\rho }_{ab}-{\rho }_{ab}{{C}_{2}}^{\dagger }{C}_{2}).$$

where *C*_2_ = (*κ*_*a*_)^1/2^*a* + (*κ*_*a*_)^1/2^*b*.

When using the quantum trajectory method^26,28^, we can write the effective Hamiltonian of coupled system as

17 $${H}_{eff}={H}_{S}-\frac{i}{2}{{C}_{2}}^{\dagger }{C}_{2}={H}_{a}+{H}_{b}-\frac{1}{2}i[{\kappa }_{a}{a}^{\dagger }a+{\kappa }_{b}{b}^{\dagger }b+2\sqrt{{\kappa }_{a}{\kappa }_{b}}a{b}^{\dagger }].$$

Cavity A is also coherently driven by a pulsed field: Ω(*t*)(*a*^†^ + *a*). And Ω_*t*_ is proportional to the amplitude of the driving pulse with Ω(*t*) = Ω_0_exp[−(*t* − *t*_0_)^2^/*τ*^2^], where *τ* is the duration of the time dependent Gaussian pulse, *t*_0_ and Ω_0_ are constants for chosen driving pulse. Then the non-Hermitian effective Hamiltonian including a coherent drive can be rewritten as refs^26,28^

18 $${H}_{eff}={H}_{a}+{H}_{b}-i/2[{\kappa }_{a}{a}^{\dagger }a+{\kappa }_{b}{b}^{\dagger }b+2{({\kappa }_{a}{\kappa }_{b})}^{\mathrm{1/2}}a{b}^{\dagger }]+{\rm{\Omega }}(t)({a}^{\dagger }+a)+\sqrt{({\kappa }_{b}/{\kappa }_{a})}{\rm{\Omega }}(t)({b}^{\dagger }+b).$$

In the Markov approximation, Eq. (18) includes the system A and B, their interaction *ab*^†^ with broken time symmetry and a coherent input. Photons can be annihilated from A and created in B. Cavity A and B are coupled through the composite collapse operator *C*_2_ = (*κ*_*a*_)^1/2^*a* + (*κ*_*a*_)^1/2^*b*. Then we simulate the system by quantum trajectory method.

### Quantum trajectory simulation

Denote the coupled system state at time *t* as $|\psi (t)\rangle$ and the Schödinger equation of the composite system is

19 $$i\frac{d|\psi (t)\rangle }{dt}={H}_{eff}(t)|\psi (t)\rangle ,$$

For a single trajectory, in a very short time interval *δt* ($\delta t\ll {\kappa }^{-1}$ and *κ* = *κ*_*a*_ = *κ*_*b*_ is the decay rate of each cavity), the system would evolve into an unnormalised state: $|\tilde{\psi }(t+\delta t)\rangle =(1-i{H}_{eff}\delta t)|\psi (t)\rangle$. And the probability that no photon decays from B in the time interval is: ${p}_{0}=\langle \tilde{\psi }(t+\delta t)|\tilde{\psi }(t+\delta t)\rangle =1-p$. And $p=\delta t\langle \psi (t)|{C}_{2}^{\dagger }{C}_{2}|\psi (t)\rangle$ presents the probability that a quantum jump takes place in *δt*. In other words, the emission times are determined in a Monte Carlo simulation using the rate function $\langle \psi (t)|{C}_{2}^{\dagger }{C}_{2}|\psi (t)\rangle$^26^.

In the simulation, we choose a random number 0 < *r* < 1 and compare *p* and *r* at the end of the time interval. If *p* < *r*, we normalize the state

20 $$|\psi (t+\delta t)\rangle =\frac{|\tilde{\psi }(t+\delta t)\rangle }{{\sqrt{p}}_{0}}.$$

Then we continue the evolution of non-Hermitian effective Hamiltonian further. Once *p* > *r*, we see a quantum jump happens and we should take renormalization

21 $$|\mathop{\psi }\limits^{ \sim }(t+\delta t)\rangle \to |\psi (t+\delta t)\rangle =\frac{{C}_{2}|\mathop{\psi }\limits^{ \sim }(t+\delta t)\rangle }{p/\delta t}.$$

where operator *C*_2_ is the collapse operator representing for the happening of quantum jumps. To simulate the one-cavity case, one just needs to take *κ*_*b*_ = 0 and no photons enters into cavity B.

The proposed scheme is shown in Fig. 1, analogy to the one-cavity case in ref.^29^. Both cavities have two mirrors on either side (left and right) and have the same nonlinear strength and decay rate: *χ* = *χ*_*a*_ = *χ*_*b*_, *κ* = *κ*_*a*_ = *κ*_*b*_. The pulse travels from the left to the right. For each cavity, the driving pulse is incident on the left mirror with high reflectivity (whose decay rate is *κ*_*left*_) and leaks out from the other (*κ*_*right*_). Since the mirrors of either resonator has decay rates ${\kappa }_{left}\ll {\kappa }_{right}\approx \kappa$, for the single mode cavity, photons will almost leak out from the right(low reflectivity) mirror. Thus a driving pulse is incident on cavity A and finally leaks out from the right side of cavity B. Ideally, as a single-photon source, single photons should leak out from cavity B every time the whole system is operated or just short period after a single pulse enters cavity A. And the collapse operator *C*_2_ = (*κ*_*a*_)^1/2^*a* + (*κ*_*a*_)^1/2^*b* to the total cavity decay is the only output channel being monitored.

Also, the output light in Fock basis should be $|{b}_{out}\rangle =\sum _{n\mathrm{=0}}^{\infty }{c}_{n}|n\rangle$, where |*c*_*n*_|^2^ is normalized representing the probability of state |*n*〉. When large numbers of trajectories are simulated, we can estimate the value |*c*_*n*_|^2^. We analyze the output light of cavity B by using an ideal single-photon detector (a simulation-based detection which does not really happen). For each single pulse put in, we monitor and count the clicks detected at the output of cavity B. When *click* = *n*, a n-photon event is detected. Specifically, for a ideal single-photon sources, no more then a single click (*click* = 1 or *click* = 0) should be detected. In the simulation, we run 6000 pulses trajectories. Then we can estimate the value |*c*_*n*_|^2^ by *P*_*n*_ = |*c*_*n*_|^2^ = *N*_*click*=*n*_/6000. The value *N*_*click*=*n*_ means there are *N* events of n-photon detection.

In the simulation, we use cavity parameters: *χ* = 15 GHz, Δ = 1 GHz. One can verify that the Kerr nonlinear coefficient *χ* with the material *SiO*_2_/*Ag* (*V*_*eff*_ = 10^−2^ *μm*^3^) can be larger than 10 *μ*eV (15.2 GHz)^17^. Besides, for Kerr materials, the nonlinear coefficient *χ* can be further increased through the reduction of cavity mode volume.

To make input pulses entering the cavity one by one, the minimum period of input pulses *f*^−1^ should be several times of the cavity linewidth *κ*^−1^. In our work, we take *f* = *κ*/5. In one-cavity case in ref.^19^ the repetition rate is only 200 MHz. One can raise *f*  by increasing *κ* to some extent. However, to make photon blockade happening, we cannot choose a too bad cavity with the value *κ* too large. To further raise the *f*, we cascade two or more cavities and lowerdown the cavity quality factors. So cascading cavities can obtain a larger *f* and stronger nonlinearity strength. In the two-cavity case, we take *κ* = 5 GHz and *κ* = 10 GHz. Compared with the one-cavity case in ref.^19^, our proposal may effectively increase the repetition rate. For each *κ* we choose, we only change the parameters of the input light: the driving amplitude Ω_0_ and pulse duration *τ*. Discussions are shown below.

To further increase the repetition rate *f*, we can utilize N cascaded cavities. In this way, one could generalize the effective Hamiltonian of the coupled N-cavity system as:

22 $${H}_{eff}=\sum _{j=1}^{N}{H}_{j}-i/2\sum _{j=1}^{N}{\kappa }_{j}{a}_{j}^{\dagger }{a}_{j}-i\sum _{j=1}^{N-1}{({\kappa }_{j}{\kappa }_{j+1})}^{1/2}{a}_{j}{a}_{j+1}^{\dagger }]+{\rm{\Omega }}(t)\sum _{j=1}^{N}[\sqrt{{\kappa }_{j}/{\kappa }_{1}}({a}_{j}^{\dagger }+{a}_{j})].$$

*H*_*j*_ stands for the free cavity mode and interactions inside the cavity *j*. Like the case when *N* = 2, *H*_1_ = *H*_*a*_ is the only cavity that is injected with pulse. The corresponding collapse operator should be ${C}_{N}=\sum _{j=1}^{N}{\kappa }_{j}^{\mathrm{1/2}}{a}_{j}$.

By defining the collapse operator *C*_*N*_ we can use the quantum trajectory method discussed above.

### Cascaded photon blockade sources

**Figure 2:**
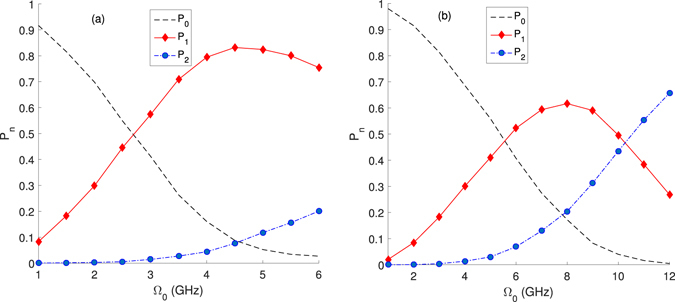
*P*_*n*_ verses Ω_0_: (**a**) when *χ* = 15 GHz, *τ* = 0.2 ns, *κ* = 5 GHz; (**b**) when *χ* = 15 GHz, *τ* = 0.1 ns, *κ* = 10 GHz.

We first analyze the photon number probability *P*_*n*_ in photon blockade with amplitude Ω_0_ when *τ* = 0.2 ns, *κ* = 5 GHz and *τ* = 0.1 *ns*, *κ* = 10 GHz. In Fig. 2, *P*_*n*_ has a strong dependence on Ω_0_ in both figures. In Fig. 2(a), it is shown that when Ω_0_ = 4.5 GHz (*P*_0_ = 8.80%, *P*_1_ = 83.18%, *P*_2_ = 7.62%, *P*_3_ = 0.40%), *P*_1_ could occupy a comparatively largest proportion at 83.18%.

**Figure 3:**
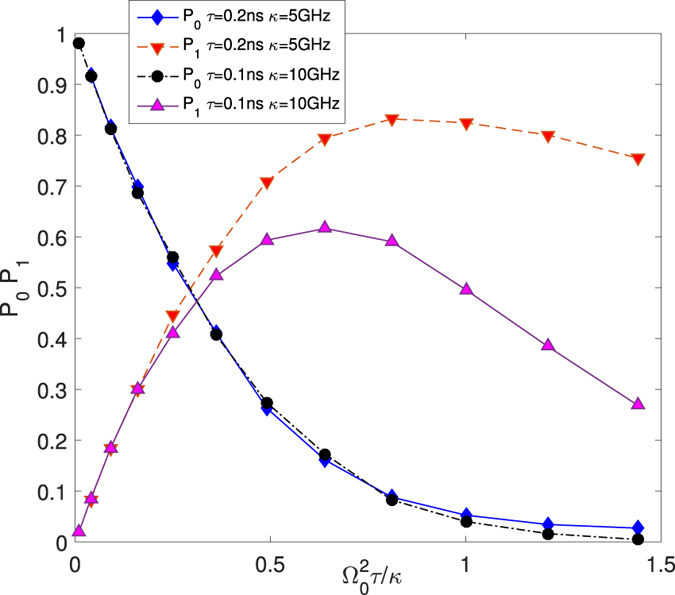
*P*_0_ and *P*_1_ verse ${{\rm{\Omega }}}_{0}^{2}\tau /\kappa$ when *τ* = 0.2 ns, *κ* = 5 GHz and *τ* = 0.1 ns, *κ* = 10 GHz.

In Fig. 2, we also notice that *P*_*n*_s at Ω_0_ = 1 GHz, *τ* = 0.2 ns,*κ* = 5 GHz are equal to to those at Ω_0_ = 2 GHz, *τ* = 0.1 ns, *κ* = 10 GHz.Thus in Fig. 3, we draw *P*_*n*_ verses ${{\rm{\Omega }}}_{0}^{2}\tau /\kappa$ when *τ* = 0.2 ns, *κ* = 5 GHz and *τ* = 0.1 ns, *κ* = 10 GHz from Fig. 2. It shows roughly the same of *P*_0_ and *P*_1_ in different chosen parameters when ${{\rm{\Omega }}}_{0}^{2}\tau /\kappa \, < \,0.5$. In weak driving photon-blockade regimes, ${{\rm{\Omega }}}_{0}^{2}\tau /\kappa$ is small to make photon blockade happening effectively. This also verifies that mean photon number $\mu =\sqrt{\pi /2}{{\rm{\Omega }}}_{0}^{2}\tau /\kappa$^19,30^. However, when ${{\rm{\Omega }}}_{0}^{2}\tau /\kappa  > 0.5$, *P*_1_ in different photon blockade systems becomes much different.

**Figure 4:**
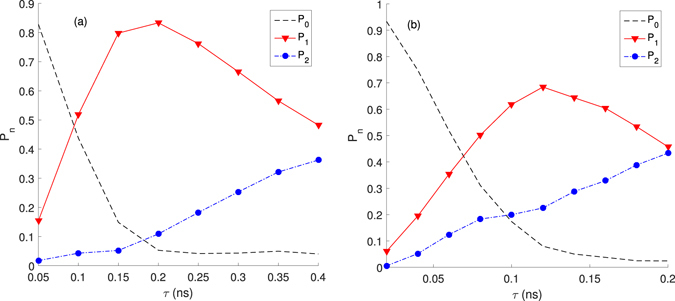
*P*_*n*_ verses *τ*: (**a**) when *χ* = 15 GHz, Ω_0_ = 5 GHz, *κ* = 5 GHz; (**b**) when *χ* = 15 GHz, Ω_0_ = 8 GHz, *κ* = 10 GHz.

It is worthy of being mentioned that the chosen values of *τ* is mainly affected by the cavity decay *κ*. We also show from Fig. 4 how *τ* affects the output light field. For example, in the left figure when *τ* = 0.2 ns, we see that *P*_1_ = 83.32% is the largest proportion among all *P*_*n*_. However, if we further increase the value of *τ*, when *τ* > 0.2 *ns* and *τ* > 0.12 ns, *P*_1_ rapidly attenuates while *P*_2_ grows remarkably. From the optimized values of *τ* (0.2 ns and 0.12 ns), we find $\mu =\sqrt{\pi /2}{{\rm{\Omega }}}_{0}^{2}\tau /\kappa \approx 1.0$ in both figures. And *μ* may provide us a useful way to optimize *P*_1_.

**Figure 5:**
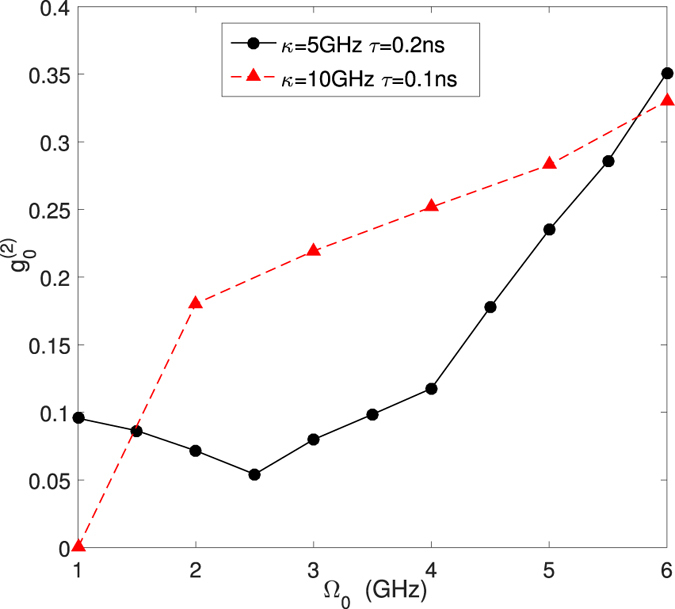
Second correlation function *g*^(2)^(0) verses Ω_0_ when *χ* = 15 GHz, *τ* = 0.2 ns, *κ* = 5 GHz and *χ* = 15 GHz, *τ* = 0.1 ns, *κ* = 10 GHz.

It may be also important to characterize the statistics properties of single-photon sources via the second-order correlation *g*^(2)^(0). We also know *g*^(2)^(0) < 1 means sub-poissonian statistics of output field. In our work, we calculate the second-order correlation *g*^(2)^(0) with different sources (see Fig. 5) using ${g}^{\mathrm{(2)}}\mathrm{(0)=}\sum _{n=0}^{\infty }n(n-1){P}_{n}/{(\sum _{n\mathrm{=0}}^{\infty }n{P}_{n})}^{2}$^30^. However, *g*^(2)^(0) cannot give us enough information about the probability of emitting one photon each time the source works. For example, for a light source with 98% vacuum state, 1.99% one-photon state and 0.01% two-photon state (*g*^(2)^(0) = 0.01). It has a considerably small *g*^(2)^(0) but very few single photons. Given the fact, in QKD, *g*^(2)^(0) is not a useful way to estimate the performance. We had better optimize *P*_1_ in the first place.

In the simulation above, the repetition rate *f* can be 2 GHz when *κ* = 10 GHz with *P*_1_ = 68.43%. However, the one-cavity case in Table 1 shows when *κ* = 10 GHz, the optimized solution is ${P}_{1}^{^{\prime} }=\mathrm{56.17 \% }$, which is much less than *P*_1_ = 68.43% (when *κ* = 5 GHz, ${P}_{1}^{^{\prime} }=\mathrm{76.57 \% } < {P}_{1}=\mathrm{83.32 \% }$). In other words, *κ* = 10 GHz is too large for a single cavity. So cascading two cavity can effectively raise the repetition rate.

**Table 1:** Optimized one-cavity photon-blockade sources (PBS) when *f* = 1 GHz,*κ* = 5 GHz and *f* = 2 GHz, *κ* = 10 GHz.

Source	*P*_0_	*P*_1_	*P*_2_	*P*_3_	*f*
source1	3.77%	76.57%	18.27%	1.37%	1 GHz
source2	13.07%	56.17%	28.85%	1.80%	2 GHz

It is needed to be mentioned that when simulating a three-cavity system, one can use our trajectory method through the corresponding collapse operator ${C}_{3}={\kappa }_{1}^{\mathrm{1/2}}{a}_{1}+{\kappa }_{2}^{\mathrm{1/2}}{a}_{2}+{\kappa }_{3}^{\mathrm{1/2}}{a}_{3}$. The nature of our cascaded source is based on the single photon blockade. So when we choose cavities with the same decay rate *κ*, a two-cavity source is natural better than a one-cavity source. For instance, when a pulse with the photon number distribution *C*_0_, *C*_1_, *C*_*n*_... pass through a single Kerr blockade cavity, the photon number distribution turns into *P*_0_, *P*_1_, *P*_*n*_ and *P*_1_ > *C*_1_, *P*_0_ > *C*_0_ because multi-photon states are suppressed. So the advantage of the two-cavity source is to further turn multi-photon states into single-photon or vacuum states. It is the same with the three-cavity system. A raise of single-photon states allows to relax the constraints on the repetition rate imposed by the cavity lifetime.

### Applications in QKD

We now apply the optimized CPBS (cascaded photon-blockade source) to decoy-state QKD. We hope to generate a higher key rate compared with the PBS (photon-blockade source without a cascade) and the optimized WCS (week coherent sources optimized key rate with infinite different intensities for decoy states). With a typical decoy-state method protocol using 3 different intensities, we borrow the results from ref.^31^ to calculate the key rates. Say, Alice randomly emits pulses from sources of density matrices: ${\rho }_{0}=|0\rangle \langle 0|$, ${\rho }_{d}={\sum }_{k=0}^{J}{a}_{k}|k\rangle \langle k|$ and ${\rho }_{s}={\sum }_{k=0}^{J}{a}_{k}^{^{\prime} }|k\rangle \langle k|$, where *a*_*k*_ ≥ 0, ${a}_{k}^{^{\prime} }\ge 0$ for all *k*, $\sum {a}_{k}=\sum {a}_{k}^{^{\prime} }=1$. Here, we call *ρ*_0_, *ρ*_*d*_ and *ρ*_*s*_ as vacuum source, decoy source and signal source respectively. Denote the counting rate of source *ρ*_0_, *ρ*_*d*_ and *ρ*_*s*_ as *s*_0_, *S*_*d*_ and *S*_*s*_. Borrowing formula (17) of ref.^31^, we can lower bound the single-photon counting rate as

23 $${s}_{1}\ge \frac{{a}_{2}^{^{\prime} }({S}_{d}-{a}_{0}{s}_{0})-{a}_{2}({S}_{s}-{a}_{0}^{^{\prime} }{s}_{0})}{{a}_{2}^{^{\prime} }{a}_{1}-{a}_{1}^{^{\prime} }{a}_{2}}.$$

So the fractions of the single-photon counts for the signal source is

24 $${{\rm{\Delta }}}_{1}^{^{\prime} }=\frac{{a}_{1}^{^{\prime} }{s}_{1}}{{S}_{s}},$$

One can calculate the final key rate for the signal source by refs^32,33^

25 $${R}_{s}={{\rm{\Delta }}}_{1}^{^{\prime} }[1-H({t}_{1})]-qH(t).$$

Here, *t*_1_ is the estimated phase-flip error rate of single-photon pulses; *t* is the observed bit-flip error rate of signal source; *q* is the factor of error correction inefficiency. And *H* is the binary Shannon entropy: *H*(*x*) = −*x*log_2_(*x*) − (1 − *x*)log_2_(1 − *x*).

**Figure 6:**
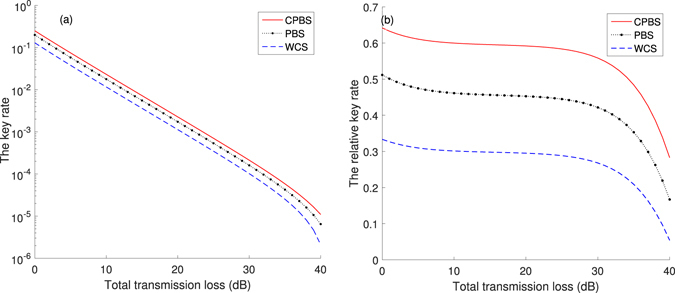
(**a**) Key rates of decoy state BB84 protocol with different sources: cascaded photon-blockade source (CPBS), the photon-blockade source without a cascade (PBS)^19^ and WCS. (**b**) The relative value of the key rates between chosen sources and the perfect single-photon source (PSPS).

In Fig. 6, we present some numerical simulations using different sources: cascaded photon-blockade source (CPBS), photon-blockade source (PBS) without a cascade and optimized weak coherent state sources (WCS). The system parameters and chosen sources (decoy sources and signal sources) used in numerical simulations are listed in Tables 2 and 3. The chosen sources have the same repetition rate *f* = 2 GHz. The single-photon probability of the PBS is low (56.17%) because we choose a too bad cavity with *κ* = 10 GHz (*f* = 2 GHz). But a CPBS allows a large *κ* with a high single-photon probability (68.43%). In Fig. 6, the key rate is raised drastically by using the CPBS at the same repetition rate. Equivalently, CPBS can raise the repetition rate.

**Table 2:** System parameters used in numerical simulations of QKD: *e*_0_: error rate of vacuum count, *e*_*d*_: misalignment-error probability; *p*_*d*_: dark count rate per detector; *q*: factor for error correction inefficiency^19^.

*e*_0_	*e*_*d*_	*p*_*d*_	*q*
0.5	1.5%	3.0 × 10^−6^	1

**Table 3:** Photon-blockade sources used in numerical simulations of QKD when *κ* = 10 GHz (*f* = 2 GHz).

*source*	*P*_0_	*P*_1_	*P*_2_	*P*_3_	*P*_4_
PBS_*decoy*_	71.53%	26.35%	2.07%	0.05%	0
PBS_*signal*_	13.07%	56.17%	28.85%	1.80%	0.10%
CPBS_*decoy*_	55.92%	41.03%	3.00%	0.05%	0
CPBS_*signal*_	8.00%	68.43%	22.52%	0.98%	0.07%

**Figure 7:**
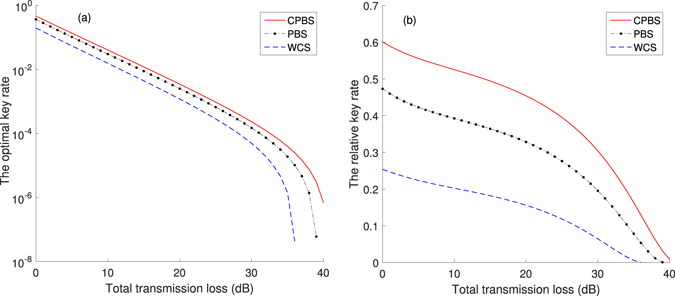
(**a**) Optimal key rates of decoy state BB84 protocol with statistical fluctuation: cascaded photon-blockade source (CPBS), the photon-blockade source without a cascade (PBS)^19^ and the WCS using three different intensities (0, 0.2 and 0.5). (**b**) The relative value of the key rates between chosen sources and the perfect single-photon source (PSPS). The data size *N* = 10^9^.

In Fig. 7, using a 3-intensity BB84 protocol, we also show the numerical simulations of the optimal key rates with statistical fluctuation^34–36^. When taking account into the statistical fluctuation, the data size *N* become the great influence to the final key rate. Thus we take *N* = 10^9^ as an example. Considering the finite-size effects, we take a failure probability of 10^−7^ with a normal distribution with parameter optimized^34–36^. Other system parameters and chosen sources (decoy sources and signal sources) can be from Tables 2 and 3. In Fig. 7, we choose WCS with three different intensities (0, 0.2 and 0.5). The simulation also shows the superiority of our proposed source.

## Discussion

We have proposed single-photon sources in cascaded Kerr photon blockade systems. The system has advantages in its controllability and flexibility compared with single-emitter systems. And the latter might have difficulties with deterministic positioning and their degree of inhomogeneity. At the output of the second cavity, we find an enhanced probability of single photon occupancy, and simultaneously a reduced vacuum and multi-photon probability, which allows to relax the constraints on the repetition rate imposed by the cavity lifetime. Parameters are optimized and *P*_1_ can be higher than 80% with very few vacuum and multi-photon states. By cascading two cavities, we effectively increase the repetition rate up to 2 GHz with *P*_1_ = 68.4% at the nonlinear strength *χ* = 15 GHZ. When the quasi sources are applied in the decoy state QKD with and without statistical fluctuation, the key rate can be both raised drastically.
