# Author Correction: Cascaded Kerr photon-blockade sources and applications in quantum key distribution

**DOI:** 10.1038/s41598-018-23428-w

**Published:** 2018-04-12

**Authors:** Ao Li, Yiheng Zhou, Xiang-Bin Wang

**Affiliations:** 1 State Key Laboratory of Low Dimensional Quantum Physics, Tsinghua University, Beijing, 100084 People’s Republic of China; 2 Synergetic Innovation Center of Quantum Information and Quantum Physics, University of Science and Technology of China, Hefei, Anhui 230026 China; 3 Jinan Institute of Quantum Technology, Shandong Academy of Information and Communication Technology, Jinan, 250101 People’s Republic of China

Correction to: *Scientific Reports* 10.1038/s41598-017-07589-8, published online 04 August 2017

The original version of this Article contained an error in the title of the paper, where the word “sources” was incorrectly given as “soruces”. This has now been corrected in the PDF and HTML versions of the Article.

